# Putative Neural Network Within an Olfactory Sensory Unit for Nestmate and Non-nestmate Discrimination in the Japanese Carpenter Ant: The Ultra-structures and Mathematical Simulation

**DOI:** 10.3389/fncel.2018.00310

**Published:** 2018-09-19

**Authors:** Yusuke Takeichi, Tatsuya Uebi, Naoyuki Miyazaki, Kazuyoshi Murata, Kouji Yasuyama, Kanako Inoue, Toshinobu Suzaki, Hideo Kubo, Naoko Kajimura, Jo Takano, Toshiaki Omori, Ryoichi Yoshimura, Yasuhisa Endo, Masaru K. Hojo, Eichi Takaya, Satoshi Kurihara, Kenta Tatsuta, Koichi Ozaki, Mamiko Ozaki

**Affiliations:** ^1^Department of Biology, Graduate School of Science, Kobe University, Kobe, Japan; ^2^National Institute for Physiological Sciences, Okazaki, Japan; ^3^Division of Biology, Department of Natural Sciences, Kawasaki Medical School, Kurashiki, Japan; ^4^Research Center for Ultra-High Voltage Electron Microscopy, Osaka University, Ibaraki, Japan; ^5^Department of Mathematics, Faculty of Sciences, Hokkaido University, Sapporo, Japan; ^6^Graduate School of Frontier Biosciences, Osaka University, Suita, Japan; ^7^Department of Electrical and Electronic Engineering, Graduate School of Engineering, Kobe University, Kobe, Japan; ^8^Department of Applied Biology, Kyoto Institute of Technology, Kyoto, Japan; ^9^Graduate School of Information Systems, The University of Electro-Communications, Chofu, Japan; ^10^Department of Biological Science, Faculty of Life and Environmental Science, Shimane University, Matsue, Japan

**Keywords:** olfactory receptor, chemosensillum, chemical communication, innexin, ant, ultra-structures, mathematical simulation

## Abstract

Ants are known to use a colony-specific blend of cuticular hydrocarbons (CHCs) as a pheromone to discriminate between nestmates and non-nestmates and the CHCs were sensed in the basiconic type of antennal sensilla (*S. basiconica*). To investigate the functional design of this type of antennal sensilla, we observed the ultra-structures at 2D and 3D in the Japanese carpenter ant, *Camponotus japonicus*, using a serial block-face scanning electron microscope (SBF-SEM), and conventional and high-voltage transmission electron microscopes. Based on the serial images of 352 cross sections of SBF-SEM, we reconstructed a 3D model of the sensillum revealing that each *S. basiconica* houses > 100 unbranched dendritic processes, which extend from the same number of olfactory receptor neurons (ORNs). The dendritic processes had characteristic beaded-structures and formed a twisted bundle within the sensillum. At the “beads,” the cell membranes of the processes were closely adjacent in the interdigitated profiles, suggesting functional interactions via gap junctions (GJs). Immunohistochemistry with anti-innexin (invertebrate GJ protein) antisera revealed positive labeling in the antennae of *C. japonicus*. Innexin 3, one of the five antennal innexin subtypes, was detected as a dotted signal within the *S. basiconica* as a sensory organ for nestmate recognition. These morphological results suggest that ORNs form an electrical network via GJs between dendritic processes. We were unable to functionally certify the electric connections in an olfactory sensory unit comprising such multiple ORNs; however, with the aid of simulation of a mathematical model, we examined the putative function of this novel chemosensory information network, which possibly contributes to the distinct discrimination of colony-specific blends of CHCs or other odor detection.

## Introduction

The natural environment surrounding living organisms is filled with chemical information, and animals have developed adaptive chemosensory systems to utilize this environmental information for purposes such as food source or mate recognition or individual identification especially in social animals. Insects have characteristic olfactory organs called sensilla, which are involved in general odor or pheromone sensing (Hallberg and Hansson, 1999; Steinbrecht, 1999; Hansson and Stensmyr, 2011; de Fouchier et al., 2017). In some cases, each type of sensilla housing multiple receptor neurons works as a sensory unit for specific biological purpose. For example, many insect species use sex pheromones for attracting mates, and their sex pheromone-sensitive sensilla, which house a few olfactory receptor neurons (ORNs), have been enthusiastically studied as simple odor sensory units (Kaissling, 1987; Haupt et al., 2010).

Social insects have evolved sophisticated chemical communication ability by means of various pheromones (Hölldobler, 1995; Vander Meer, 1998; Ozaki et al., 2005; Mizunami et al., 2010; Nick and d'Ettorre, 2012; Ozaki and Hefetz, 2014; Sharma et al., 2015; Leonhardt et al., 2016). In an ant a colony, worker ants use antennation, a typical behavior of contact investigation with antennae, to accept nestmates but reject conspecific non-nestmates and hetero-specific worker ants. In many ant species, worker ants utilize a colony-specific blend of cuticular hydrocarbons (CHCs) as a social pheromone for nestmate recognition (Vander Meer, 1998; Lahav et al., 1999; Ozaki et al., 2005; Brandstaetter et al., 2008; Guerrieri and d'Ettorre, 2008; Guerrieri et al., 2009; Nick and d'Ettorre, 2012; Ozaki and Hefetz, 2014). Worker ants have species-specific composition of different CHC combinations with different components among species, but they have common colony-specific CHC blends with the same components within a species. In *C. japonicus*, worker ants from different colonies have the colony-specific CHC blends comprising 18 species-specific CHCs (Ozaki et al., 2005). Ozaki et al. (2005) were the first to study the chemosensory system for nestmate vs. non-nestmate discrimination in *C. japonicus* and proved that the *Sensilla basiconica* (*S. basiconica*) function as CHC sensilla. Within each *S. basiconica* of *C. japonicus*, which were later discovered to be female-specific (Nakanishi et al., 2009), more than 100 ORNs extend the dendritic processes (Ozaki et al., 2005). The receptor membranes of ORNs are surrounded by chemosensory protein (CSP)-containing sensillar lymph, allowing lipophilic CHCs to be transported by CSP to the receptor membranes of ORNs (Ozaki et al., 2005; Hojo et al., 2015). In the early electrophysiological recordings in *C. japonicus, S. basiconica* were stimulated by contact with CHCs that were scattered in a CSP-containing aqueous solution (Ozaki et al., 2005). It was reported that the number of *S. basiconica* responding to nestmate CHCs was significantly smaller than that responding to non-nestmate CHCs, and similar results were found in other ant species (Kidokoro-Kobayashi et al., 2012). In a later study, when stimulated by the vapor of heated CHCs, *S. basiconica* of *Camponotus floridanus*, for example, responded to not only non-nestmate CHCs but also nestmate CHCs (Sharma et al., 2015), and it was suggested that there are morphologically similar but functionally different subtypes of *S. basiconica* on the antennae of *C. floridanus*. Therefore, it is considered that the *S. basiconica* of ant probably classified into subtypes would be multifunctional olfactory organ not only for nestmate and non-nestmate discrimination but also for other hydrocarbon or general odor sensing as in other insects (Kropf et al., 2014; Couto et al., 2017).

In several species, *S. basiconica* ORNs project into a distinct antennal lobe region consisting of a cluster of the same number of glomeruli (Kelber et al., 2010; Nishikawa et al., 2012; McKenzie et al., 2016; Couto et al., 2017). While, the functional properties and sensory mechanism of *S. basiconica* involved in nestmate recognition in ants have been studied, they are not yet fully understood because of its complexity with many ORNs, expressing specific olfactory receptor (OR) genes, respectively. Because of this complexity we suspect that there are some information filtration or modification systems within *S. basicomica*. Presumably, those ORNs do not behave like independent parallel cables but may functionally connect and influence each other.

In the present paper, we showed the beaded-structures along the dendritic processes and documented in detail about their number and localization. The beaded-structure looked to provide a platform for functional connection among ORNs via close apposition of membranes. However, it was difficult to conduct proper experiments to prove functional modification in the response of a *S. basiconica*, which was expected by hypothesizing functional connection among ORNs. Thus, we examined a simplified mathematical simulation for the inter-dendritic neural network based on the cable theory and proposed possible modification of its responsiveness to virtual stimulation.

## Materials and methods

### Ants

Worker ants of the Japanese carpenter ant, *C. japonicus*, were collected from around the nests of several colonies on the Kobe University campus. Nestmates from each colony were transferred into a plastic box (23 × 16 × 8 cm^3^) with a small artificial nest box (5 × 7.5 × 1.8 cm^3^) covered with a red plastic sheet and maintained at room temperature in our laboratory for several days or weeks until use. Ants were fed a synthetic diet (Dussutour and Simpson, 2008) and had freely access to water. We also obtained a whole nest with a queen, hundreds of workers, and some virgin queens and males; the nest was transferred to Kawasaki Medical School, where we prepared sample blocks for electron microscopy. Thus, the collected ants were reared in an artificial field comprising a foraging yard (23 × 16 cm^2^) and an artificial nest with several plastic chambers (5 × 7.5 × 1.8 cm^3^) connected by plastic tubes (8 mm inner diameter) in Kawasaki Medical School. Ants were fed a diet of diluted maple syrup and mealworms with water.

### Electron microscopy

The protocol for serial block-face scanning electron microscopy (SBF-SEM) was adopted from Deerinck et al. (SBEM Protocol v7_01_2010; https://ncmir.ucsd.edu/sbem-protocol). *C. japonicus* workers were anesthetized on ice for 10 min, and the antennae were detached from the head and cut into pieces. The specimens were fixed in 2.5% glutaraldehyde and 2% paraformaldehyde in 2 mM CaCl_2_-containing 0.1 M cacodylate buffer (pH 7.4) for 18 h at 4°C. After washing in the same buffer containing CaCl_2_, specimens were osmicated in 2% osmium tetroxide in 0.1 M cacodylate buffer (pH 7.4) containing 1.5% potassium ferrocyanide for 2 h at 4°C. The specimens were then washed with distilled water and placed in Millipore filtered thiocarbohydrazide (TCH) solution for 20 min. After that, the specimens were fixed in 2% osmium tetroxide in distilled water for 1 h at room temperature. They were re-washed with distilled water and then incubated in 1% uranyl acetate (aqueous) for 18 h at 4°C. After washing with distilled water, the specimens were treated with Walton's lead aspartate staining for 60 min at 60°C. Next, 15-min-wash in distilled water was three times repeated, and dehydration was performed with a series of 50, 70, 90, 100%, and again 100% ethanol for 15 min, respectively. Specimen were then soaked in propylene oxide for 20 min at room temperature. Specimens were then transferred into a 1:1 mixture of epoxy resin (49.6% LUVEAK812; 21.8% DDSA; 26.7% MNA; 2.0% DMP30, nacalai Tesque, Kyoto, Japan) and propylene oxide and maintained on a slow speed rotator in a draft chamber at room temperature overnight. The next day, the antenna pieces were gently rotated in 100% epoxy resin for 3 h at room temperature and embedded in 100% epoxy resin and incubated at 60°C for 48 h. The sample block in the epoxy resin was carefully trimmed to obtain a single *S. basiconica* and was used for three types of electron microscopy: SBF-SEM, conventional transmission electron microscopy (TEM), and ultra-high-voltage electron microscopy (UHV-EM). It was not difficult to discriminately find *S. basiconica* on the *C. japonicus* antenna under a stereomicroscope (SZX9 Olympus) by its characteristic outer cuticular structure. Since it is known that there are no other types of antennal sensilla but only *S. basiconica* houses more than 100 ORNs (see Nakanishi et al., 2009), every *S. basiconica* chosen as the electron microscopic specimen had been confirmed by counting the number of ORNs within the sensillum. However, in *C. japonicus*, it is not clear whether there are sub-types of *S. basiconica* on the antenna and either morphological or functional discrimination among sub-types. Hence we did not discriminately choose our specimen among putative subtypes of *S. basiconica*.

Using SBF-SEM Gatan 3view (Gatan, Inc., CA, USA)-Zeiss ΣIGMA/VP & MARLIN (Carl Zeiss Microscopy GmbH, Jena, Germany), serial block-face images of an *S. basiconica* were obtained at 1.2 kV accelerating voltage. We aligned the serial images and loaded the digital data onto the image processing Amira software program (Indeed Visual Concepts GmbH, Berlin, Germany; TGS Inc.). The dendritic process areas selected on each cross image were manually segmented. Using the surface rendering method, the data were reconstructed into a 3D structural model of a unit of the dendritic process within a sensillum.

We also obtained ultra-thin sections of *S. basiconica* for conventional TEM observation using JEM-1400 (JEOL Ltd., Tokyo, Japan) at an accelerating voltage of 80 kV and 2 μm-thick sections for UHVEM observation using H-3000 (Hitachi Co., Tokyo, Japan) at an accelerating voltage of 2,000 kV. For observation by UHVEM, the 2 μm-thick sections were mounted on Formvar-coated slot grids, immersed in 3% uranyl acetate in 70% methanol, heated in a microwave oven for 30 s, incubated for 10 min at room temperature, and rinsed with distilled water. The sections were then immersed in SATO lead stain solution, heated in a microwave oven for 30 s, incubated for 10 min at room temperature, and rinsed with distilled water. The sections were covered using an additional Formvar membrane and coated on both sides with evaporated carbon, followed by studding with 20 nm gold particles. Images were taken at 20,000 × from −60° to +54°C at 2°C intervals around a single axis and captured a resolution of 4096 × 4096 pixels at a pixel size of 0.85 nm using 486BK CCD camera (Hitachi Co., Tokyo, Japan). Each set of tilted images were aligned using gold particles as fiducial markers and reconstructed using Simultaneous Iterative Reconstruction Technique (SIRT) (Gilbert, 1972). All tomograms were analyzed using IMOD software (Kremer et al., 1996). 3D models were drawn by tracing membranous structures using “3DMOD,” which is the graphics component of IMOD.

### Blast and HMM search for innexin family

We used BLASTp to search the protein CDS references of *C. japonicus* (Hojo et al., 2015) for innexin candidates using protein sequences of *Drosophila melanogaster* innexin (inx1 to inx8) with an e-value cutoff of 1.0E−15. We also performed a HMM search using the innexin superfamily (pf00876) as a query.

### Western blot of antennal proteins for CjapInx3

Antennae were detached from 50 *C. japonicus* worker ants that were anesthetized on ice and were immediately frozen in liquid nitrogen in a hand mortar. The samples were then homogenized with 200 μl of SDS-polyacrylamide gel electrophoresis (PAGE) sample buffer and used for western blot analysis with the anti-CjapInx3 antiserum raised against a specific epitope (LGIDEGERRYHS) of innexin 3 of *C. japonicus* (see **Figure 4**). Five ants equivalent extract in 20 μl of sample buffer was loaded per lane for SDS-PAGE. After electrophoresis, proteins were transferred from the gel to polyvinylidene difluoride (PVDF) membranes (immobilon-P; Merck KGaA, Darmstadt, Germany). PVDF membranes were blocked with Blocking One (NACALAI TESQUE, INC., Kyoto, Japan) at room temperature for 2 h, incubated with anti-CjapInx3 antiserum (1:100 dilution with Can Get Signal; TOYOBO CO., LTD., Osaka, Japan) at room temperature for 1 h, and processed using the Vectastain ABC kit (Vector Laboratories, Burlingame, CA, USA) according to manufacturer's instructions. The CjapInx3 signal was detected using Chemi-Lumi One Super (NACALAI TESQUE, INC., Kyoto, Japan).

### Immunohistostaining of antennal sections with anti-CjapInx3 antibody

Antennae detached from cold anesthetized *C. japonicus* worker ants were cut into small pieces and immediately fixed in a solution of 1% paraformaldehyde, 0.25% ZnCl_2_, 127 mM NaCl, and 3.5 mM sucrose at 4°C overnight. After fixation, the antennae were twice incubated in ant ringer solution (4.8 mM TES, 127 mM NaCl, 6.7 mM KCl, 2 mM CaCl_2_, and 3.5 mM Sucrose) with 30% sucrose at 4°C twice for 1 h and incubated overnight at 4°C. Subsequently the antennae were embedded in O.C.T. compound (Sakura Finetek Japan Co., Ltd., Tokyo, Japan) and frozen in a cryostat (CM1850; Leica Biosystems Nussloch GmbH, Wetzlar, Germany) to prepare 8 μm-thick vertical sections. The obtained sections were mounted on glass slides. The slides were washed in acetone at −20°C for 30 min, dried for 1 h, washed 3 times in ART (ant ringer solution with 0.05% Triton-X100, 5 min per time), and then activated by incubation in HistoVT One (NACALAI TESQUE, INC., Kyoto, Japan) at 70°C for 20 min. After being washed 3 times in ART, the slides were treated with Blocking One (NACALAI TESQUE, INC., Kyoto, Japan) at room temperature for 2 h and incubated either with rabbit antisera against CjapInx3 (for test) or with pre-immune rabbit sera (for control) (1:300 diluted with Can Get Signal solution B; TOYOBO CO., LTD., Osaka, Japan) overnight at 4°C. The next day, the sections were washed 4 times in ART (5 min each time) and incubated with Alexa 594-conjugated goat anti-rabbit IgG (A11012; Thermo Fisher Scientific, Wilmington, DE, USA, 1:800 diluted with Can Get Signal solution B) overnight at 4°C. Subsequently the slides were washed four times in ART and mounted with Fluoromount (K024; DIAGNOSTIC BIOSYSTEMS, Pleasanton, CA, USA). The slides were stored in the dark at 4°C until microscopic observation. We acquired the fluorescence images of *S. basiconica*, using a super-resolution microscope system (N-SIM; Nikon Corporation, Tokyo, Japan) coupled to an ECLIPSE Ti2-E inverted microscope (Nikon Corporation, Tokyo, Japan) with a 60× water immersion objective lens (SR Plan Apo IR 60×, NA1.27; Nikon Corporation, Tokyo, Japan), LU-N3-SIM laser unit (Nikon Corporation, Tokyo, Japan), and ORCA-Flash 4.0 sCMOS camera (Hamamatsu Photonics K.K., Hamamatsu, Japan). The excitation wavelength was 561 nm, and the emission was filtered using a 605 nm filter. Fluorescence images of the longitudinal (0.64 μm thickness) and cross sensillar sections (6 μm thickness) were reconstructed with NIS elements AR software (Nikon Corporation, Tokyo, Japan). Differential interference images of the longitudinal sensillar sections were observed using a FV1000 confocal microscope (Olympus Corporation, Tokyo, Japan) coupled to a BX61W1 microscope (Olympus Corporation, Tokyo, Japan) with a 60× water immersion objective lens (UPLSAPO60XW, NA 1.20; Olympus Corporation, Tokyo, Japan).

In order to reduce non-specific signals, we tried to examine this immunohistostaining experiment under various conditions in blocking, washing and anti-serum treatment, but it was difficult to completely avoid the non-specific signals. If we sonicated the specimen in the fixative, non-specific staining tended to be reduced. However, thus the sonication treatment increased in risk of breaking sensilla, hence we stopped the sonication treatment. Observation with different excitation wavelengths was not helpful to clarify the outline shape of sensillaum, but we could recognize the outline of the cuticular shaft of the sensillum in the 3D reconstructed fluorescent images by rotating it using NIS elements AR software (Nikon Corporation, Tokyo, Japan), which is useful to get all directional optical views. Moreover, using this software, fluorescent signals within a sensillum were discriminately seen from those on the outer surface of the sensillum.

### Mathematical simulation

All simulations were performed using the Julia environment (version 0.6.1). A mathematical model of 10 or 20 cables with mutual connections via GJs, which is based on cable theory (Rall, 1959; Segev et al., 1995; Koch, 1999), was used in the simulations (detailed mathematical expressions are given in the Appendix). Each cable consists of nine serially-connected passive compartments in the distal part of dendritic process and one active compartment as an approximal part with a spiking (impulse generating) mechanism (Morris and Lecar, 1981). GJs are assumed to connect passive compartments among different cables. Hypothesizing that the current influx is generated at the most distal parts of the dendritic processes of the ORNs, an external input current is given to one end of the passive compartments (the very top compartments corresponding to the distal part of the dendritic processes) in each cable, and an active compartment with an impulse generating mechanism is connected to the other end of the passive compartments.

## Results

### Serial block-face scanning electron microscope observations: fine interior structure of *Sensilla basiconica*

**Figure 1:**
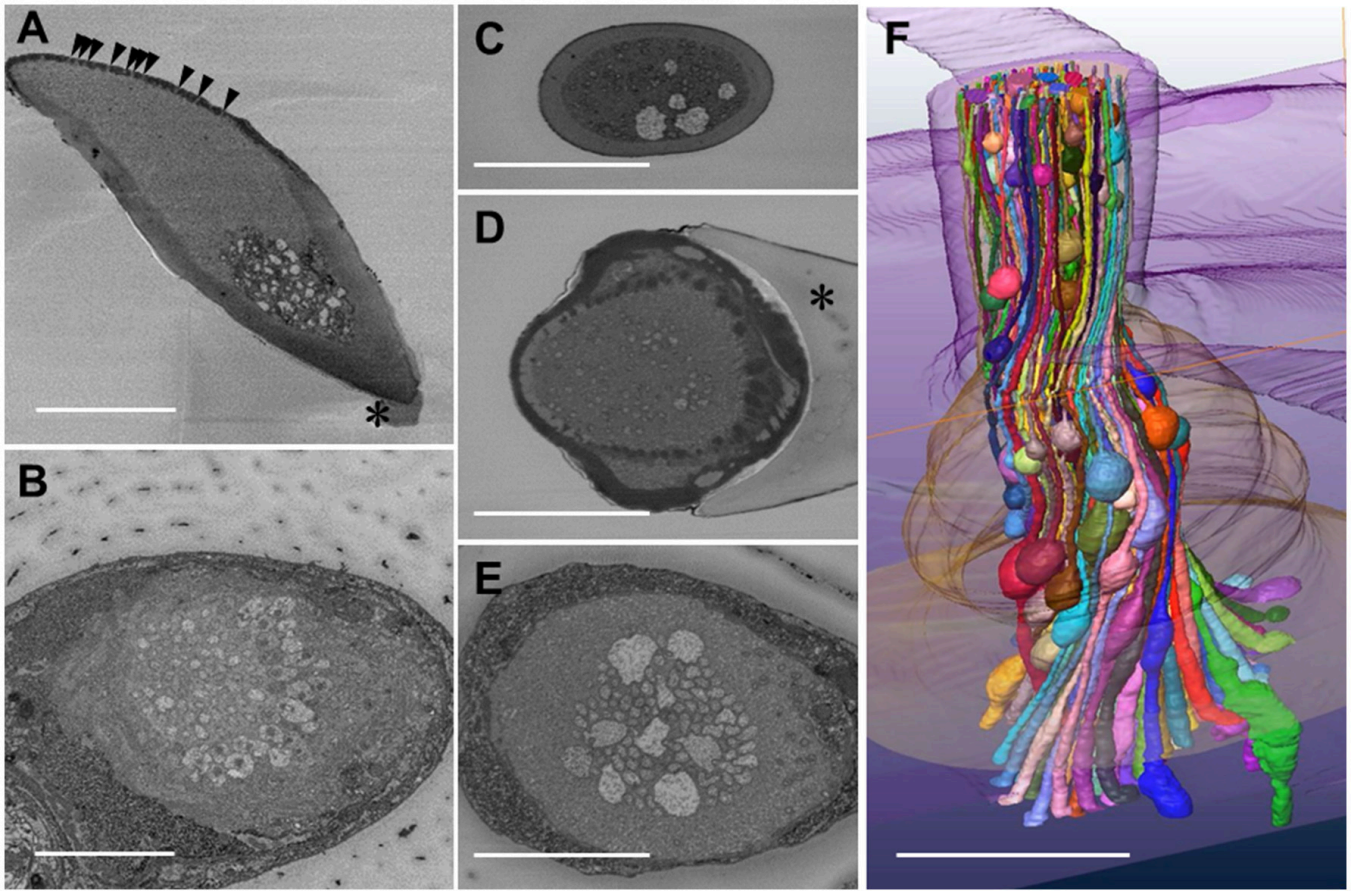
Serial block face scanning electron microscope images and reconstruction of a three-dimensional structure of olfactory receptor neurons' dendritic processes in a *Sensilla basiconica* of *Camponotus japonicus* worker ant. **(A)** A longitudinal oblique section of the *S. basiconica*. Arrowheads and asterisk indicate openings of olfactory pores and a part of the socket cuticle at the basement of the sensillar shaft, respectively. **(B)** A cross image of the *S. basocinica* with 102 dendritic processes at the junction with the inner segments of ORNs. **(C–E)** The cross images of *S. basiconica* at 6 and 1 μm distal and 4 μm proximal from the outer surface of the antennal cuticle (see arrowheads of **Figure 6A**). **(F)** 3D-structural model of a bundle of color-coded dendritic processes within a sensillum reconstructed by 352 cross images at 70 nm interval (24.6 μM in total length). Bars indicate 5 μm.

We got the SBF-SEM images from nine specimens of *S. basiconica*, and the dendritic processes with beaded-structures were seen in all of them, although the beaded-structures could not precisely be compared in their number or localization among those specimens. Thus, we show the representative data in Figure 1. Figure 1A presents a longitudinal oblique section of *S. basiconica*, which includes the sensillar top with olfactory pores (arrowheads) and a small piece of the socket cuticle at the basement of the sensillar shaft (asterisk). The thickness of the cuticular wall is not uniform around the perimeter. Around the sensillar top, one side, where the multiple openings of the olfactory pores are seen, is thinner than the other side. Beneath those openings, there is a cell-free space that no dendritic processes can reach; by contrast, many dendritic processes are seen from the middle to the basement of the sensillum (Figures 1B–E).

Using the best prepared specimen, we obtained fine serial cross images of *S. basiconica* under SBF-SEM, starting at 12 μm from the top of the sensillum where we could sufficiently adjust the focus on the membranes of the dendritic processes. In total, 596 cross images were serially obtained from the distal to proximal region at 70 nm intervals until all the dendritic processes were outside the visual field. Figure 1B shows an example cross image with 102 dendritic processes, some of which show connective cilia at the junctions with the inner segments of ORNs' dendritic processes. Figures 1C,D are cross sections of a sensillum at 6 and 1 μm distal and Figure 1E at 4 μm proximal from the outer surface of the antennal cuticle, respectively, (see arrowheads in **Figure 6A** where the outer surface of the antennal cuticle is at zero level). Despite variations among cross images, all sections along this *S. basiconica* constantly revealed 102 dendritic processes. Almost all cross sections revealed thin dendritic processes; however, Figures 1C,E show 5 and 9 dendritic processes with large cross-sectional areas, respectively. Using 352 images of all 596 serial sections obtained in the representative sensillum, we reconstructed the 3D structure model over 24.6 μm along the sensillar shaft (Figure 1F). As expected, the 102 dendritic processes, which form a moderately twisted bundle and are housed in a sensillum, had no branches but had characteristic beaded-structures, which could result in the large cross-sectional areas shown in Figures 1C–E.

### Transmission electron microscope observations: morphological evidence of adhesion between olfactory receptor membranes

**Figure 2:**
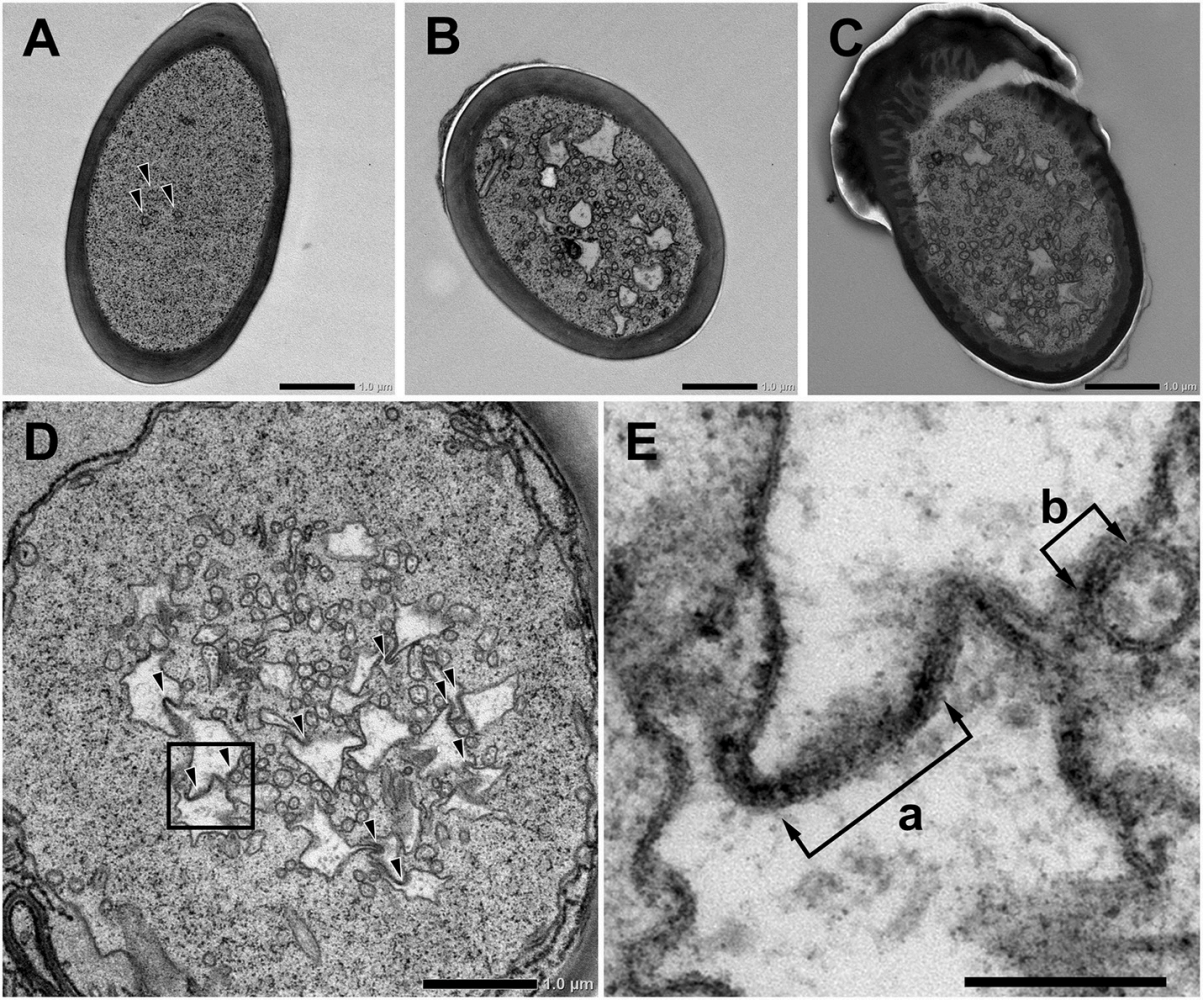
Transmission electron microscope images of *Sensilla basiconica* and adhesion between olfactory receptor membranes. **(A–C)** Cross images of ultra-thin sections of an *S. basiconica* of *C. japonicus* worker ant. Three sections are at the middle of the sensillar shaft, where few dendritic processes are seen **(A)**, more proximal **(B)** and the level of basal socket, where many dendritic processes are seen **(C)**. **(D)** A cross section beneath the antennal cuticular surface. **(E)** High magnification image of a square of **(D)**, showing adhesion between adjacent membranes (double-headed arrows). Bars indicate 1 μm in **(A–D)** and 200 nm in **(E)**.

**Figure 3:**
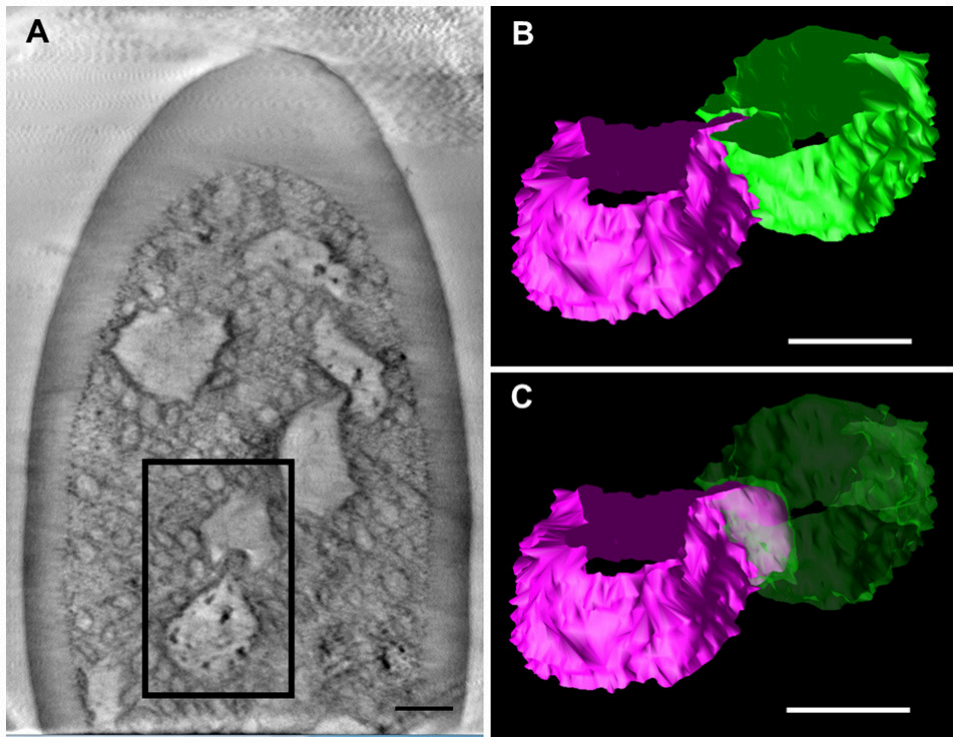
Tomographic analysis of beaded-structures in a *Sensilla basiconica*. **(A)** A tomographic slice image of 3.4 nm-thick in an *S. Basiconica* of *C. japonicus* worker. **(B)** 3D tomographic models of two adjacent beads, surface of which are colored in magenta and green, in a square of **(A)**. **(C)** Same as **(B)**, but one bead surface is shown as translucent green. Bars indicate 50 nm.

We further observed the cross sections of *S. basiconica* using TEM. As shown in Figure 1A, there is a cell-free space at the top of the sensillum. Figure 2A is a cross section of the middle of the sensillum, where only a few small cross sections of the dendritic processes are observed (arrowheads), Figure 2B is more proximal, and Figure 2C is at the basal socket. The cross images of TEM in Figure 2 are consistent with those of SBF-SEM in Figure 1. At the beads of the dendritic processes, where large cross-sectional areas were observed, we frequently found the closely adjacent cell membranes of dendritic processes (arrowheads in Figure 2D). Figure 2E is a high magnification image of the square area in Figure 2D. At a bead a dendritic process closely adheres to the adjacent membranes of the beady (double-headed arrow a) and non-beady parts of the neighboring dendritic processes (double-headed arrow b) in Figure 2E. In addition, we show UHV-EM image focusing on an adhesion region between beads of adjacent dendritic processes (square area in Figure 3A), in which the membrane of one bead (magenta in Figures 3B,C) is invaginated into the other (green in Figures 3B,C).

### Super-resolution fluorescent microscopy observations: localization of CjapInx3

**Figure 4:**
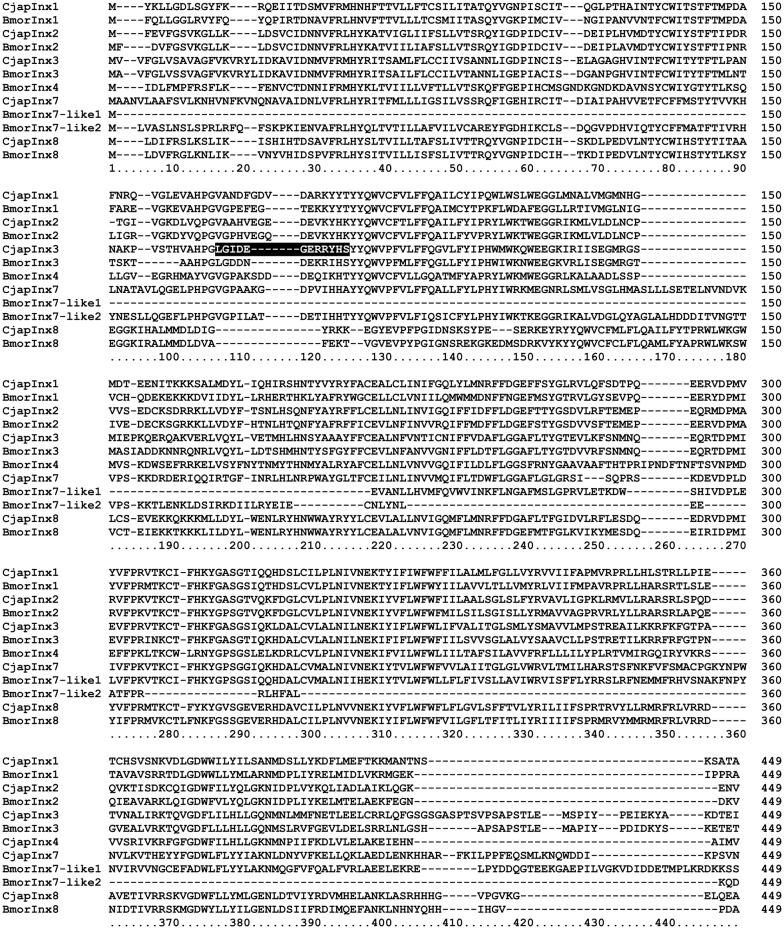
Amino acid sequence alignment of innexin molecules expressing in the antenna of *Camponotus japonicus*. Subtypes of innexin expressing in *C. japonicus* antennae (CjapInx1, CjapInx2, CjapInx3, CjapInx7, CjapInx8) are compared with each other and with *Bombyx* innexins in amino acid sequence. A sequence used as an epitope for immunohistostaining is highlighted.

Based on the results of RNA sequencing analysis (Hojo et al., 2015), we found that *C. japonicus* expresses five innexin subtypes, namely CjapInx1, 2, 3, 7, and 8, as putative GJ proteins in the antennae (Figure 4). We prepared anti-CjapInx3 antiserum against an amino acid sequence in an extracellular loop of CjapInx3, which was chosen as the specific epitope (white characters in Figure 4).

**Figure 5:**
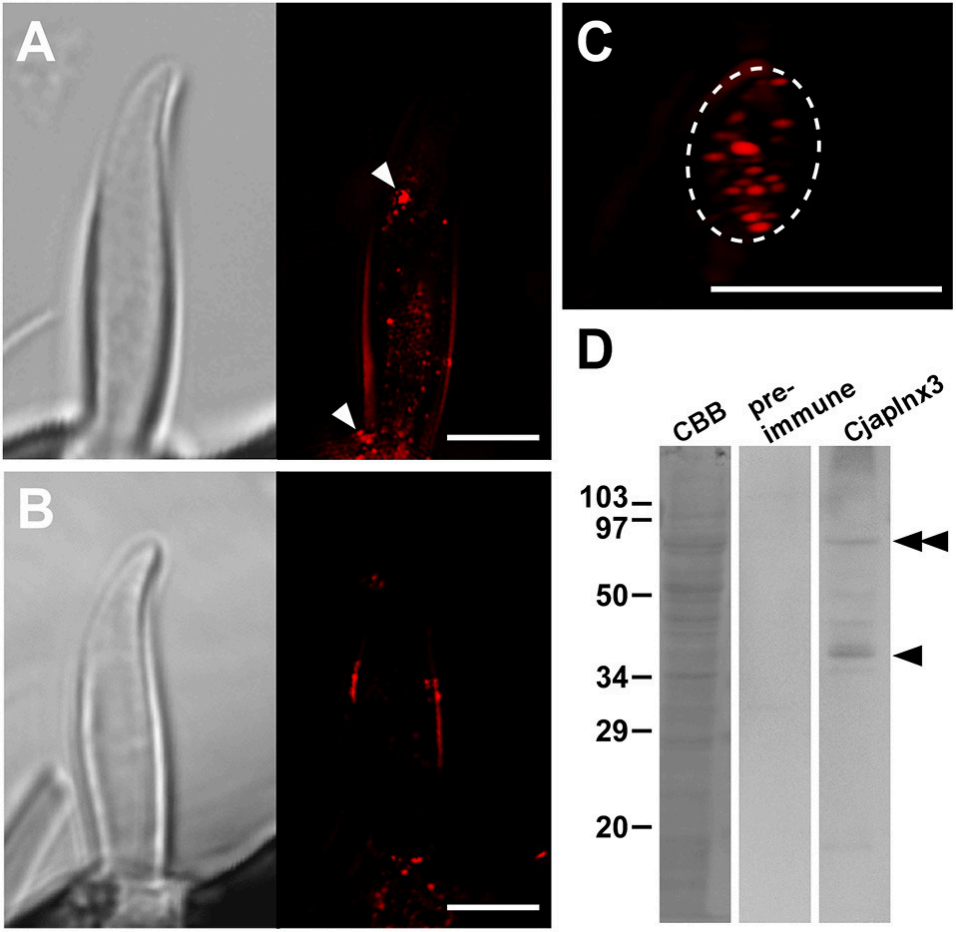
Immunohistostaining for CjapInx3 in the *Sensilla basiconica*. **(A)** Differential interference image (Left) and fluorescence image of the longitudinal section of test sensillum treated with anti-CjapInx3 antiserum (Right). **(C)** A fluorescence image of the cross section of the test sensillum. Inner perimeter of the sensillar cuticle wall is traced with broken line. **(B)** Differential interference image (Left) and fluorescence image of the longitudinal section of control sensillum treated with pre-immune serum (Right). Bars indicate 5 μm. **(D)** Western blot of the *C. japonicus* antennal proteins with anti-CjapInx3 antiserum used for the immunohistostaining. Arrowhead and double arrowhead indicate monomeric and dimeric CjapInx3.

Using anti-CjapInx3 antiserum, we localized this innexin subtype in the antennae of *C. japonicus*. Totally, 19 sensilla were examined, however 10 of them were useless; 6 were inconveniently oriented and 4 had no dendritic processes in empty sensillar shafts. Consequently, 9 sensilla, which were precisely observed, exhibited inside staining with anti-CjapInx3 antiserum. Three of them and one control are shown as the supplementary material (Supplementary Figures 1A–C), and the representative images of test and control are shown in Figure 5. Figure 5A shows the representative high resolution immunohistostaining images of *S. basiconica* using anti-CjapInx3 antiserum; **Left**, differential interference image; **Right**, fluorescence image. Figure 5B shows the representative high resolution immunohistostaining images using pre-immune serum; **Left**, differential interference image; **Right**, fluorescence image. In Figure 5A **Right**, fine fluorescent spots were distributed in the proximal half of the sensillar shaft but not in the distal half. Figure 5C shows a 6-μm thick cross-sectional view of the *S. basiconica*. The dotted staining is found only inside of the sensillum (the inner perimeter of the sensillar cuticle wall is traced with a broken line), which implies that CjapInx3 is localized inside the proximal half of the *S. basiconica*. Conversely, Figure 5B **Right**, a control image hardly exhibits such fine fluorescent dotted staining as Figure 5A **Right**. Relatively strong fluorescent signals (arrowheads in Figure 5A **Right**) were sometimes seen on the outer surface of the sensillum even in the distal half (see Supplementary Figures 1A–C). Those signals are presumed to be unavoidable and non-specific (see section Materials and Methods). Nevertheless, by investigating every fluorescent image data with NIS elements AR software (Nikon Corporation, Tokyo, Japan) to get all directional optical view, we could suggest that specific signals exist in the sensillar lumen of all nine test sensilla. Such inside signals appeared more proximal than 11.84 ± 0.99 μm (average ± standard error, *n* = 9) from the top of the sensillum. In the representative immunohistostaining data shown in Figure 5A **Right**, we succeeded to measure fluorescence intensity distribution of the inside signal along the longitudinal axis of the sensillar shaft (see Supplementary Figure 1).

We also confirmed that the anti-CjapInx3 antiserum used for immunohistostaining labeled a band of 37 kDa apparent molecular mass in the western blot of antennal proteins (arrowhead in Figure 5D). Another labeled band of a higher molecular mass may correspond to the dimeric CjapInx3 (double arrowheads in Figure 5D). The pre-immune serum yielded no labeling. Given the above immunohistostaining results and this western blotting data (Figures 5A Right, 5D), we estimated that CjapInx3 is localized inside of the *S. basiconica*, overlapping the area occupied by the dendritic processes.

### Distribution of “beads” of dendritic processes

**Figure 6:**
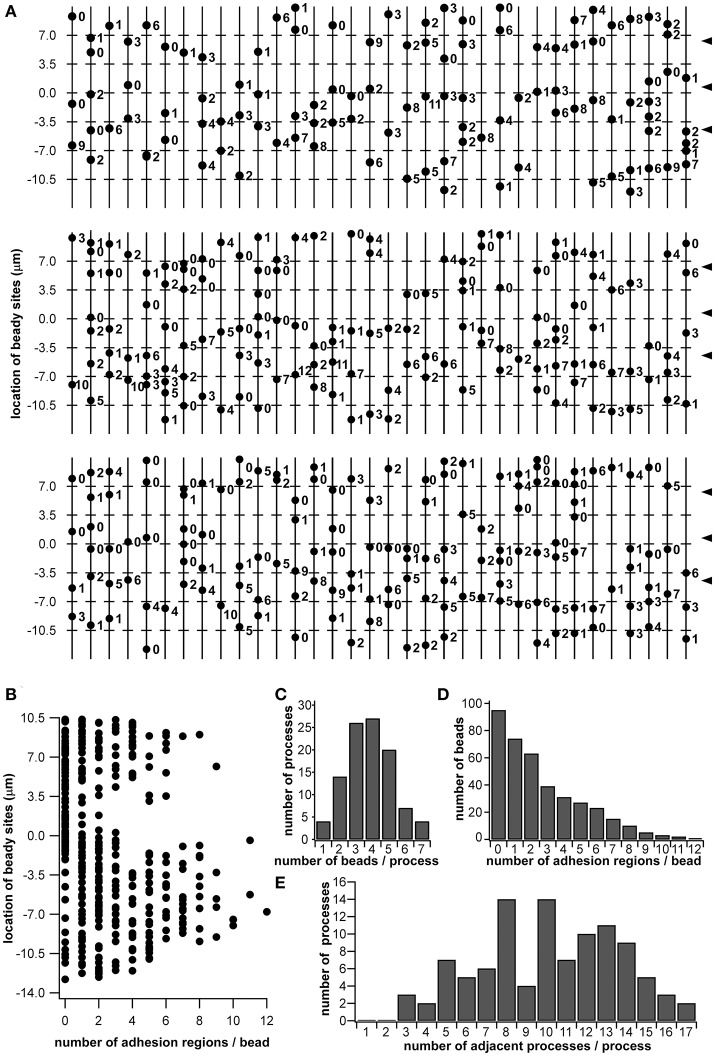
Schematic diagram of beaded-structures in the dendritic processes of ORNs and the bead distribution within a *Sensilla basiconica*. Based on the SBF-SEM images in Figure 1, beady sites and putative GJ regions on them were counted by hands. **(A)** Schematic image of beaded-structures on all of 102 dendritic processes of the ORNs within an *s. basiconica*. **(B)** Distribution of beady sites along the dendritic processes with the number of adhesion regions as putative GJs at every bead. **(C)** Distribution of the dendritic processes in the number of beads per process. **(D)** Distribution of the beads in the number of adhesion regions per bead. **(E)** Distribution of the dendritic processes in the number of adjacent processes per process.

Using the morphological data obtained via SBF-SEM (Figure 1), we enumerated all the beads in the beaded-structures of the 102 dendritic processes, identifying every adhesion region between cell membranes. In the observed range, 388 beads and 696 adhesion regions were enumerated. In Figure 6A, the 102 vertical lines with dots indicate the dendritic processes with beads. The number by the dot indicates how many adhesion regions occur at the corresponding bead. The axis of the ordinates in Figures 6A and **6B** indicates the regional level along the sensillar shaft as the distance from the outer surface level of the antennal cuticle. In Figure 6B, each dot corresponds to a bead distributed on the dendritic process, which is represented by the axis of the ordinate, while the axis of the abscissa indicates the number of adhesion regions at each bead. Thus, the distribution of the number of adhesion regions has two peaks, distal, and proximal, from the outer surface of the antennal cuticle. However, around the level of the basal socket, at a slightly distal level from the outer surface of the antennal cuticle, there are a small number of beads having no or a few adhesion regions. The distal and proximal peaks include 191 and 505 adhesion regions, respectively. When the fluorescence intensity distribution plot based on an immunohistostaining data using anti-CjapInx antiserum (Figure 5A **Right**) is superimposed on Figure 6B, both distributions indicating localization of CjapInx3 and appearance of beaded-structure are similar to each other, showing the distal peak, middle trough, and rising to the proximal peak (Supplementary Figure 1F). Figure 6C shows that every dendritic process has 1–7 beads, and the mean number of beads is four. Ninety-five of the 388 beads had no adhesion regions, but the remaining had 1–12 adhesion regions, as shown in Figure 6D. Every dendritic process is adjacent to 3–17 other dendritic processes. as shown in Figure 6E.

### Simplified mathematical model for electric connections among dendritic processes

**Figure 7:**
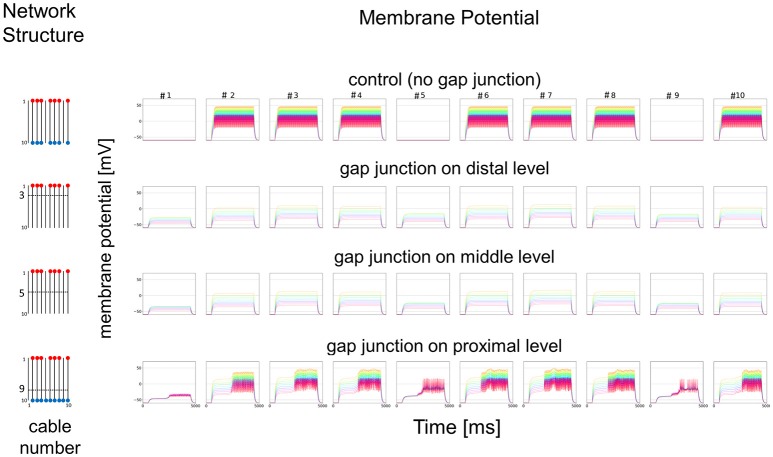
Simplified model and functional simulation with a strong stimulation. Simulation results using multi-compartment model consisting of ten cables with mutual connections via GJs. **Left**, Network structure used in simulations. Four cases with different patterns of GJs are considered (no GJs, GJs at distal, middle, and proximal levels). Horizontal broken lines in network structure indicate electric connections between different cables via GJs. Red and blue dots indicate cables being given inputs and generating impulse outputs, respectively. **Right**, Spatiotemporal responses obtained from simulation. Each row shows time courses of membrane potentials for corresponding network structure. Membrane responses at 10 different compartments in each cable are shown by colored solid lines in each subfigure. Ten differently colored solid lines (orange, yellow, chartreuse, green, cyan, azure, blue, purple, pink, and red line) show time courses of membrane potentials at corresponding compartments (compartments 1–10), respectively. Namely, orange line corresponds to the compartment at the most distal level where input is given, while red line corresponds to the compartment at the most proximal level where impulse generating mechanism is assumed.

**Figure 8:**
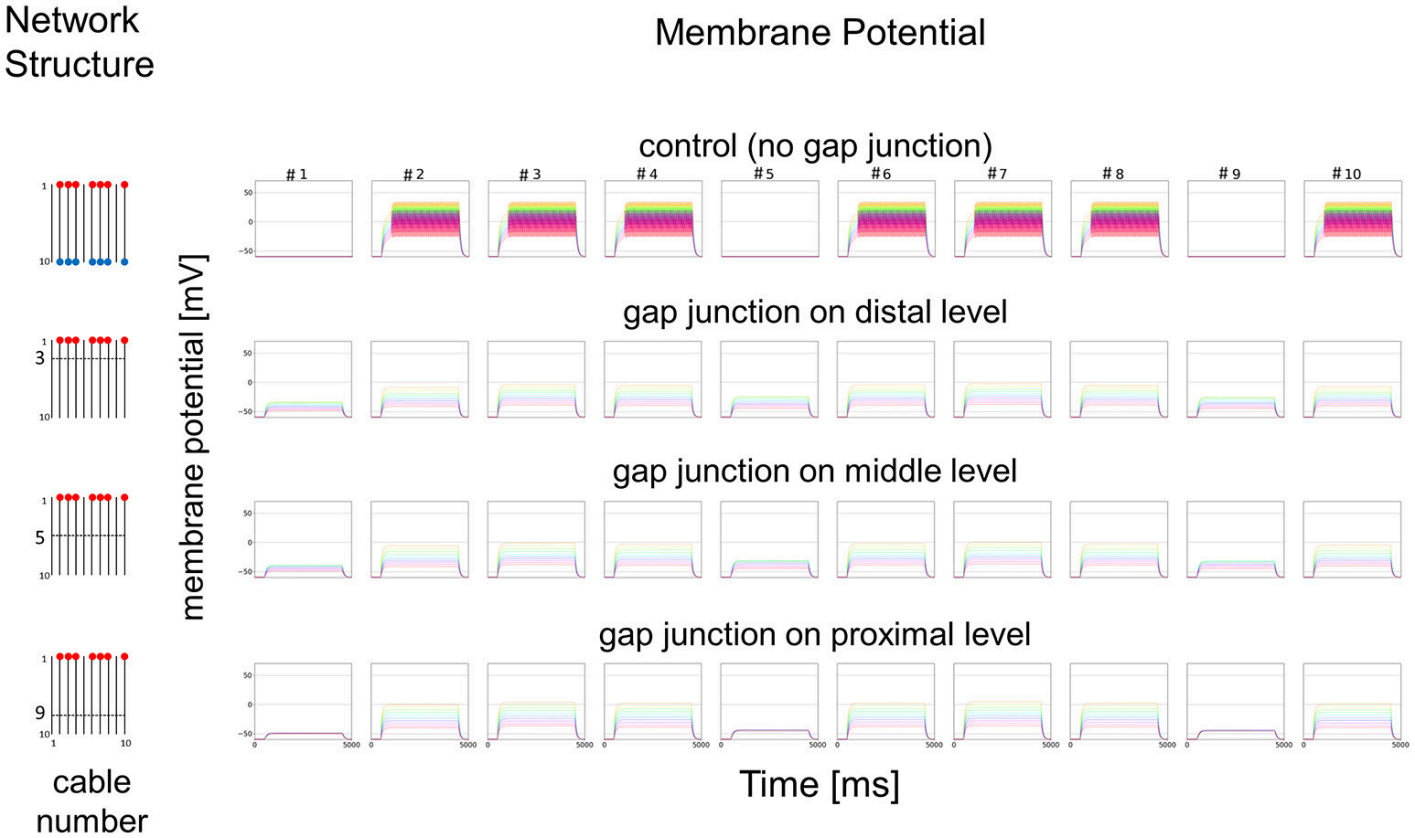
Simplified model and functional simulation with a weak stimulation. Simulation results for the small amplitude of input current in the same model as Figure 7.

As a simplified model, we first hypothesized that a set of cables (#1–10) corresponding to 10 dendritic processes existing in parallel, not three dimensionally like inside a sensillum, but two-dimensionally (see left columns in Figures 7 and 8). The cables were hypothesized to have lateral connections to neighboring cable(s) via GJs. Using a system of ordinary differential equations (A1)–(A3) based on cable theory (see Appendix), we computed the direction of propagation of the current in our compartment model described in the Materials and Methods section. When we give input current to the most distal compartments of a limited number of cables, the inward current will passively propagate along the stimulated cables and the electrically connected neighboring cables. The most proximal compartment can generate impulses, only when the inward current is larger than a threshold at the impulse generating site. Such a large inward current, even if it propagates also to the neighboring cables via electric connections, evokes impulse generating activity. In cases where no impulse appears, the inward current decreases to less than the threshold during passive propagation. Whereas in cases where impulses are generated at the most proximal compartment, back propagating firing of impulse discharge from the most proximal compartment occurs at different passive compartments along the concerned cables.

In Figure 7, we show different results in accordance with the location of the connections among cables via electric connections. More precisely, if there is no electric connection at all (see control of Figures 7,8), the external input current given in 7 cables independently contributes to the appearance of impulses in each of the 7 cables, as shown in the top row of Figure 7. However, if there are electric connections at the distal and middle levels in the second and third rows of Figure 7 (electric connections at the distal and middle levels, respectively), the impulses completely disappear, even if the amplitude of the input current is exactly the same as that of the control and the input current should evokes the same change in membrane potential as the control, because the inward current reaching the impulse generating site of each concerned cable is under the threshold level. Moreover, if the cables are connected at the proximal level (close to the impulse generation site at the basement of the dendritic processes or near soma, i.e., active compartment), then the number of cables generating impulses at their active compartments increases to 9. We also examined a different situation by decreasing the amplitude of the input current. Figure 8 shows such an amplitude that no impulse appears even if connections do exist among cables, whereas if there are no connections among cables in control, impulses are still observed in the 7 cables with the input current.

**Figure 9:**
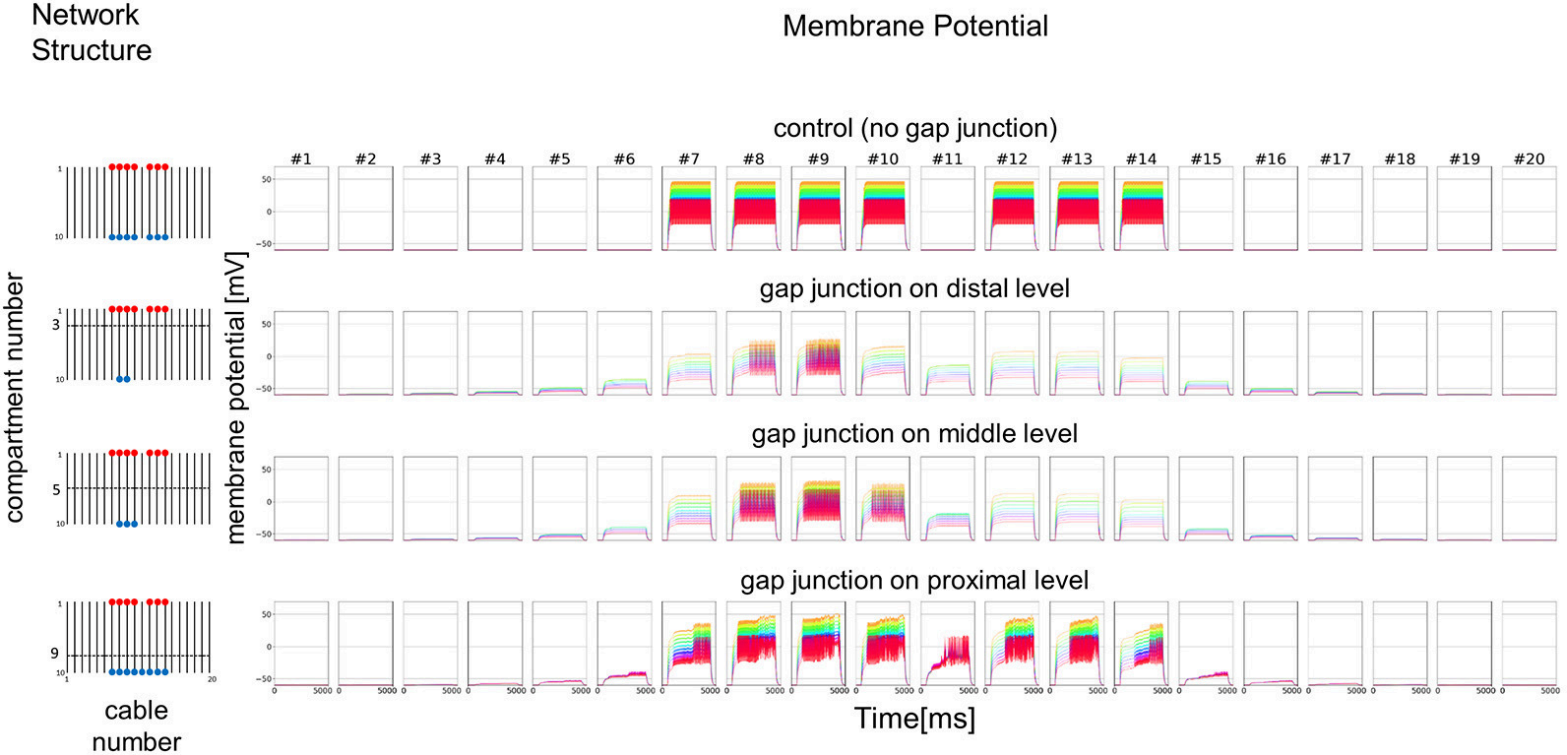
Simplified model with double number of cables and functional simulation with a strong stimulation. Simulation results using multi-compartment model consisting of 20 cables with mutual connections via GJs. Four cases with different patterns of GJs are considered in the same model as Figure 7. The stimulation with the same amplitude as in Figure 7 is applied.

We further examined the similar 2D simulation to Figure 7 but with 20 cables (#1–20), seven of which are stimulated (Figure 9). Then, impulse generation is depressed with electric connections at the distal (two of 20 cables generate impulses) or middle level (three of 20 cables generate impulses). If the cables are connected at the proximal level, however, the number of cables generating impulses at their active compartments increases to 8 including a cable that has no direct input. Given these simulations hypothesizing ORN clusters formed by the dendritic network, we suggest in such ORN clusters that electric connections at distal or middle level will decrease, and that those at proximal will increase the number of cables generating impulses at their active compartments. Thus, the ORN clustering by electric connection along dendritic processes can differently modulate the total responsiveness of a sensillum possessing multiple ORNs.

## Discussion

### Filtration or modification of olfactory information

The *S. basiconica* on the antenna of ants is a small but complicated chemosensory unit. Its complexity is dependent on the repertoire of related OR genes belonging to the 9-exon subfamily (Engsontia et al., 2015; Zhou et al., 2015; McKenzie et al., 2016; Pask et al., 2017; Slone et al., 2017), which are expressed in the *S. basiconica* ORNs projecting the same number of glomeruli in a specific antennal lobe region called T6 (Zube et al., 2008; Kelber et al., 2010; Nakanishi et al., 2010; Nishikawa et al., 2012; Kropf et al., 2014; McKenzie et al., 2016; Couto et al., 2017). Olfactory information from *S. basiconica*, when sent to the higher brain, is further processed or memorized and contribute to regulation of behaviors, some of which may be cast- or sex-specific (Zube and Rosslea, 2008; Brandstaetter and Kleineidam, 2011; Brandstaetter et al., 2011; Nishikawa et al., 2012). In this stream, olfactory information of nestmate CHCs as well as non-nestmate CHCs, when sent to the higher brain via antennal lobe, would be memorized. Then the memory of the nestmate odor could be updated as the nestmate CHC pattern is gradually changed and thereby used as a template for the nestmate recognition (Brandstaetter and Kleineidam, 2011; Brandstaetter et al., 2011; Nishikawa et al., 2012; Ozaki and Hefetz, 2014).

In addition, a possibility of filtration or modification of olfactory information at the peripheral level is considered in the present paper. In the peripheral system of *S. basiconica* in *C. japonicus*, indeed, more than 100 dendritic processes extended into the cuticular shaft (Ozaki et al., 2005). One of our unexpected findings regarding the fine structure of the sensillum was the cell-free space occupying the distal half of the inside cavity beneath the olfactory pores (Figure 1 A). This space is filled with sensillar lymph dissolving CjapCSP (Ozaki et al., 2005; Hojo et al., 2015). Although this space is open to outer environmental chemicals through olfactory pores, the aquatic sensillar lymph surrounding the receptor membranes prevents lipophilic chemicals from freely diffusing to the receptor membranes. However, CjapCSP, a carrier protein for lipophilic compounds like CHCs, allows them to reach the receptor membranes. Thus, this space may function as the first filter for stimulus chemicals. There were no structural passages crossing the sensillar lymph to the receptor membranes, like the pore tubules reported in sex pheromone-sensitive sensilla of moths (Steinbrecht, 1999). Hence, CHC-CjapCSP complexes can only reach the receptor membranes by diffusion migration. The body surfaces of worker ants, including the antennae, are covered with colony-specific CHC blends that play a role in nestmate recognition (Wang et al., 2016). Hojo et al. (2015) in their paper on RNAseq analysis in the antenna of *C. japonicus* showed that there are at least two different CSPs in *S. basiconica* and suggested that they have different function for carrying different ligands within a sensillum. This may be concerned with subtype formation and complex or multifunctional sensory mechanism in the *S. basiconica*.

Since CHCs can penetrate the cell-free space of *S. basiconica* through olfactory pores, self-CHCs in complex forms with CjapCSP should always be present in the same ratio as that on the antennae (see Supplemental Material of Ozaki et al., 2005), which could desensitize a portion of ORNs or their receptor membranes to self-CHC components in a CHC composition ratio dependent manner. According to a putative explanation by Ozaki et al. (2005) and Ozaki and Hefetz (2014), the ORNs responsible for reception of self-CHC components are desensitized more strongly than other ORNs within *S. basiconica*. Thus, the cell-free space may function as a second filter, allowing the passage of hetero-specific or non-nestmate CHC information by olfactory signal transduction more efficiently than that of nestmate CHCs or self-CHCs.

Moreover, we proposed electric connections among dendritic processes probably via GJs in the *S. basiconica* of *C. japonicus*, which may be involved in olfactory information modification in this type of sensillum. In fact, we lack direct evidence by electrophysiological experiments or dye coupling between neighboring ORNs because of technical difficulty in handling ORNs in the small cuticular apparatus of insect sensillum. However, our collected morphological data (Figures 1-3, 5) support the existence of the micro-network within the *S. basiconica*; Figure 1 shows the overall shape of the 102 dendritic processes in a bundle of characteristic beaded strings; Figures 2 and 3 indicate a 2D image and 3D surface model of adhesion between membranes of dendritic processes at the beads, respectively, and Figure 5 together with Supplementary Figure 1 suggests that CjapInx3 localizes in the proximal half of the sensillar cavity of *S. basiconica*, showing the consistent distribution with the beady site localization overlapping the dendritic process extensions (Figure 6B).

As for the innexin expression in the *C. japonicus* antennae, Hojo et al. (2015) reported that there are 4 other types of innexin than CjapInx3 (Figure 4). We have not successfully constructed specific antibodies against all of them, but against CjapInx2, CjapInx3 and CjapInx8, and have got some immuno-cytological data that can preliminarily suggest that CjapInx2 and CjapInx8 localize to other regions than the inside of the sensillar shaft in the *S. basiconica*, to which CjapInx3 seems to localize (Figure 5 and Supplementary Figure 1). As the cellular components in the inside cavity of the sensillar shaft, there are only dendritic processes of the ORNs without any supportive cells, which surround the cell bodies of ORNs at the basement of the sensillum under the antennal cuticular layer (Figures 1-3). Moreover, there are few intracellular membranous structures except for invaginations of cell membranes of the dendritic processes. As the visible intracellular structures, there are many microtubules along the dendritic processes and connective cilia at the basement (Figure 1). There might be vesicles for membrane turnover, but they were not frequently seen. Therefore, we suppose that membranous proteins like innexin, if immunohistologically detected inside of the sensillar shaft, probably locate on the cell membranes of the dendritic processes.

To convince that CjapInx3 is localized to the beaded-structures on the dendritic processes within a *S. basiconica* of *C. japonicus* and that it forms GJs, further immunohistological investigation at the electron microscopic level and functional studies of electrophysiology are required. However, given the structural evidence for apposed membranes, one could say that structural data suggests GJs could play a role and modeling demonstrates some potential implications of coupling. A simple but essential mathematical model (Figures 7, 8) suggested that such a micro-network of connecting multiple cables mimicking dendritic processes might function as a stronger-input-spread (Figure 7) or weaker-input-cut filter (Figure 8) under particular conditions.

### Putative function of micro-network in *S. basiconica* suggested by mathematical simulation

The beading of dendritic processes itself is a phenomenon also reported in the sex pheromone-sensitive sensilla of moths (Keil, 1984a,b, 1989). Furthermore, it has been directly observed under a light microscope that beads occur and even move on living dendritic process (Williams, 1988). Therefore, the possibility of beading being a fixation artifact has already been ruled out. However, the biological function of beads remains elusive. No one has proposed its potential role for network communication among ORNs in any species. Nevertheless, our study animal was different and the investigated sensillum, namely *S. basiconica* of ants, is a much more complicated olfactory sensory unit than a sex pheromone-sensitive sensillum of moths. Previous findings of beaded-structures along the dendritic processes were not on such a large scale as the *S. basiconica* in *C. japonicus*. Thus, the present paper is the first report taking up the beaded-structure that may be involved in modification of olfactory dependent neuronal/behavioral responses.

Considering putative role of the beaded structures on the nestmate and non-nestmate discrimination of ant, mathematical simulation mimicking dendritic processes, which can function as a stronger-input-spread (Figure 7) or weaker-input-cut filter (Figure 8), is suggestive. As mentioned above, it could be supposed that ORNs in the *S. basiconica* are exposed to self-CHCs continuously secreted on the antennal cuticle surface, and thus the ORNs might be more strongly desensitized to nestmate CHCs than to non-nestmate CHCs. Because of the difference in desensitization effect, input current evoked by nestmate CHCs was expected be weaker than that by non-nestmate CHCs. Moreover, in our micro-network structure simulation, weaker input current like a desensitized input current evoked by nestmate CHCs tends to result in decreased chance of impulse generation in any concerned dendritic processes as shown in Figure 8, whereas less-desensitized or stronger input current tends to result in increased chance of impulse generation as shown in Figure 7. Our mathematical model can result thus the function, only when the parallel cables mimicking the dendritic processes are connected at the proximal level close to the impulse generation site. Less-desensitized or stronger input current like receptor currents generated by the non-nestmate CHCs can still trigger impulses not only in directly stimulated ORNs but also in neighboring ORNs (Figure 7). By contrast, weaker input in previously desensitized ORNs, after being divided into neighboring ORNs, is reduced under the threshold in all concerning ORNs. However, as was reported by Brandstaetter and Kleineidam (2011), nestmate CHCs still activated the AL glomeruli, this kind of peripheral filtration would not completely function, when the nestmate CHC stimulation quantitatively overcame desensitization to the self-CHCs. However, such a stronger-input-spread or weaker-input-cut filter, when combined with the desensitization mechanism in sensory system, could be useful for sensitive detection of unusual or novel odors.

In the present study, we precisely enumerated the number of putative adhesion regions between dendritic processes in an *S. basiconica* and showed their distribution along all dendritic processes (Figure 6). In that sample, there were indeed a small number of adhesion regions at the level of the outer surface of the antennal cuticle. In contrast, a larger number of adhesion regions, as many as 505, were enumerated in the proximal part and as many as 191 in the distal part. Moreover, as shown in Supplementary Figure 1F, the distribution pattern of adhesion regions (Figure 6B) is similar to the distribution pattern of fluorescence intensity along the sensillar shaft in an example of immunohistological staining using anti-CjapInx3 antisera (Figure 5A **Right**). This supplementary data suggests involvement of GJs in the dendritic network formation in *S. basiconica* of *C. japonicus*.

Besides the electric connection by GJ forming electric synapse, in fact, ephaptic coupling has been known to be able to explain inhibitory and enhancing effects on neighboring ORNs housed in a insulated narrow space like a sensillum as a significant determinant of the olfactory code (White et al., 1990; Su et al., 2012; Van der Goes van Naters, 2013; Chen, 2015; Miriyala et al., 2018). Therefore, also in the case of *S. basiconica* of ant, ephaptic coupling effect should not be ignored. Nonetheless, in comparison with ephaptic coupling previously reported in chemosensilla of other insect species, there might be some advantage to GJ connection in its potential of flexible change of connective site. The GJ network would be flexible and such a plastic state is difficult to experimentally follow, but in the *S. basiconica*, functional connective site might properly be appeared or disappeared to form different shapes of dendritic networks within an appropriate set of ORNs, depending on age, sex, cast or social task. Based on electrophysiological data, Sharma et al. (2015) suggested that there are different types of *S. basiconica* on the antennae of *C. floridanus*, and that different combination of OR genes are expressed in different subtypes. We presume that those different *S. basiconica* subtypes could also have different shapes of dendritic networks depending on their roles, respectively.

In the present study, we demonstrated ultrastructure in *S. basiconica* of *C. japonicus*. It included characteristic beaded structure, which looked like a platform for Cell-Cell interaction. Our morphological study was limited to step into functional insight on that structure. To compensate experimental limitation, we examined mathematical simulation and found putative function as an olfactory information modifier. Yet, we need more convincing morphological data by immuno-electron microscopy and electrophysiology to certify olfactory information modification, which was suggested by mathematical simulation.

## Author contributions

YT: SBF-SEM, TEM, UHV-EM; TU: High resolution microscope, Innexin work; NM, KM: SBF-SEM, 3D model; KY: SBF-SEM, TEM; KI: UHV-EM; NK: Tomography analyses; TS: TEM; HK, JT, and TO: Mathematical model; RY and YE: Innexin work; MH: Innexin gene analysis; ET and SK: EM Image analysis; KT and KO: Western blot; MO: Supervising.

### Conflict of interest statement

The authors declare that the research was conducted in the absence of any commercial or financial relationships that could be construed as a potential conflict of interest.
