# Supplementary material for: Putative Neural Network Within an Olfactory Sensory Unit for Nestmate and Non-nestmate Discrimination in the Japanese Carpenter Ant: The Ultra-structures and Mathematical Simulation
Source: Front Cell Neurosci. 2018 Sep 19;12:310. doi: 10.3389/fncel.2018.00310 (PMC6157317; doi:10.3389/fncel.2018.00310)
Supplement: Supplementary file 1 [file Image_1.pdf]

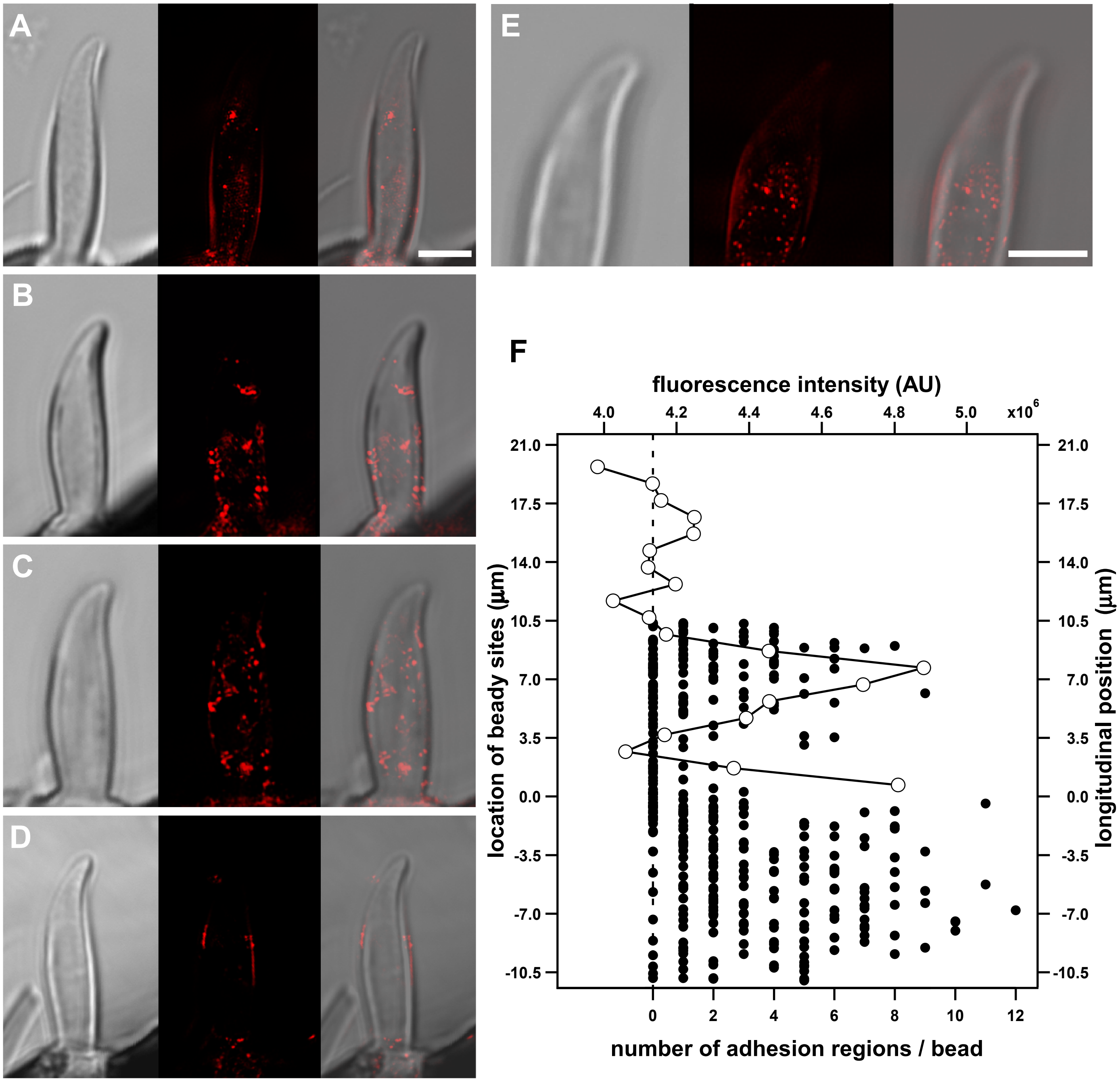

Supplementary Figure 1 Immunohistostaining for CjapInx3 in the *Sensilla basiconica* and distribution of inside signals. (A-C) Differential interference images **(Left)**, fluorescence images of the longitudinal sections of three test sensilla treated with anti-CjapInx3 antiserum **(Middle)** and the superimposed images of differential interference and fluorescence images, respectively **(Right)**. **(D)** Differential interference image **(Left)** and fluorescence image of the longitudinal section of control sensillum treated with pre-immune serum **(Middle)** and the superimposed image of both **(Right)**. **(E)** Additional data of a truncated sensillum; Differential interference image **(Left)**, fluorescence image of the longitudinal section treated with anti-CjapInx3 antiserum **(Middle)** and the superimposed image of both. Bars indicate 5 µm. **(F)** Distribution of inside signal along the sensillar shaft. Based on a fluorescent image of the sensillum **(A)**, the fluorescence intensity (horizontal axis) was measured in every $1 \times 1 \times 0.64$ µm^3 rectangular compartment stacking along the longitudinal axis of the sensillar shaft and plotted at the longitudinal position (vertical axis). This plot (open circles) is superimposed on the plot of the distribution of beady sites along the dendritic processes (closed circles) (**Figure 6B**). The average fluorescence intensity measured in the compartments more distal than 12.5 µm from the sensillar top (average $\pm$ standard error = $4.14 \times 10^6 \pm 0.02 \times 10^6$, n = 11) is fit to zero on the horizontal axis of **Figure 6B** as the background intensity, because in those 11 compartments, there are no dendritic processes extended by the ORNs.
