# Supplementary material for: Putative Neural Network Within an Olfactory Sensory Unit for Nestmate and Non-nestmate Discrimination in the Japanese Carpenter Ant: The Ultra-structures and Mathematical Simulation
Source: Front Cell Neurosci. 2018 Sep 19;12:310. doi: 10.3389/fncel.2018.00310 (PMC6157317; doi:10.3389/fncel.2018.00310)
Supplement: Supplementary file 2 [file Presentation_1.pdf]

APPENDIX

Here, we describe the mathematical model used in the simulations. As shown in **Figure 1**, we consider a system consisting of N cables with connections via gap junctions. To account for the spatial structure of the neural system, we formulate a multi-compartment model based on cable theory (Rall, 1959; Segev et al., 1995; Koch, 1999).

As shown in **Figure 1**, each cable consists of M compartments; $M-1$ compartments (from compartment 1 to compartment $M-1$) are passive, whereas compartment M is active with a spiking mechanism. These M compartments within a cable are serially connected.

We assume that only compartment 1 of each cable receives an external input. The membrane potential of compartment 1 in the nth cable, $V_{n,1}$, is assumed to obey the following differential equation:

$$C\frac{dV_{n,1}}{dt} = -g_{\mathrm{L,p}}\left(V_{n,1}-E_{\mathrm{L}}\right) - g_{\mathrm{axial}}\left(V_{n,1}-V_{n,2}\right) + I_n(t) \qquad \text{(A1)}$$

where the first term $g_{\mathrm{L,p}}\left(V_{n,1}-E_{\mathrm{L}}\right)$ is a leak current, the second term $g_{\mathrm{axial}}\left(V_{n,1}-V_{n,2}\right)$ is an axial current between compartments 1 and 2, and the third term $I_n(t)$ is an input current. Here, C is membrane capacitance. $g_{\mathrm{L,p}}$ and E_{L} indicate the leak conductance of passive compartments and reversal potential of the leak current, respectively. g_{axial} is the axial conductance.

The membrane potential in other passive compartments, $V_{n,i}(i \in \{2,3,\dots,M-1\})$, are assumed to obey the following differential equation:

$$C\frac{dV_{n,i}}{dt} = -g_{\mathrm{L,p}}(V_{n,i} - E_{\mathrm{L}}) - g_{\mathrm{axial}}(V_{n,i} - V_{n,i-1}) - g_{\mathrm{axial}}(V_{n,i} - V_{n,i+1})$$

$$- g_{\mathrm{gap}} \sum_{(m,j)\in\aleph(n,i)} (V_{n,i} - V_{m,j}) \qquad \text{(A2)}$$

where the fourth term $g_{\mathrm{gap}} \sum_{(m,j)\in\aleph(n,i)} (V_{n,i} - V_{m,j})$ shows the electrical synaptic currents via gap junctions between different cables. Here, $\aleph(n,i)$ shows a set of compartments in other cables that have a connection with compartment i in nth cable. The fourth term $g_{\mathrm{gap}} \sum_{(m,j)\in\aleph(n,i)} (V_{n,i} - V_{m,j})$ shows the electrical synaptic currents via gap junctions between different cables. Here, $\aleph(n,i)$ shows a set of compartments in other cables that have a connection with compartment i in the nth cable.

For all cables, compartment M is assumed to have a spiking mechanism as follows:

$$C\frac{dV_{n,M}}{dt} = -g_{\mathrm{L,a}}(V_{n,M} - E_{\mathrm{L}}) - g_{\mathrm{Ca}} M_\infty(V_{n,M})(V_{n,M} - E_{\mathrm{Ca}}) - g_{\mathrm{K}} N_n (V_{n,M} - E_{\mathrm{K}})$$

$$- g_{\mathrm{axial}}(V_{n,M} - V_{n,M-1}) \qquad \text{(A3)}$$

where the second and third terms correspond to calcium and potassium currents, respectively, in the neuronal model proposed by Morris and Lecar (1981). Leak conductance of the active compartment is denoted by $g_{\mathrm{L,a}}$, maximal conductances of calcium and potassium currents are indicated by g_{Ca} and g_{K}, respectively, and their reversal potentials are indicated by E_{Ca} and E_{K}. Here, $M_\infty(V)$ in the calcium current

is a function of the channel variable expressed by the following equation:

$$M_\infty(V) = \frac{1}{2}\left(1 + \tanh\left(\frac{V - v_1}{v_2}\right)\right) \tag{A4}$$

where v_1 and v_2 are constants. N_n is a channel variable for the potassium current. The channel variable N_n obeys the following differential equation:

$$\frac{dN_n}{dt} = -\frac{N_n - N_\infty(V_{n,M})}{\tau(V_{n,M})} \tag{A5}$$

where $N_\infty(V)$ and $\tau(V)$ are functions of membrane potential as follows:

$$N_\infty(V) = \frac{1}{2}\left(1 + \tanh\left(\frac{V - v_3}{v_4}\right)\right) \tag{A6}$$

$$\tau(V) = \frac{1}{\varphi}\operatorname{sech}\left(\frac{V - v_3}{2v_4}\right) \tag{A7}$$

where v_3, v_4, and φ are constants.

In this study, we set the numbers of cables and compartments in each cable as $N = 10$ and $M = 10$, respectively. Parameter values for reversal potentials and those related to channel variables $M_\infty(V)$ and N_n are set as the values proposed by Morris and Lecar (1981) as follows: $E_{\mathrm{L}} = -60, E_{\mathrm{Ca}} = 120, E_{\mathrm{K}} = -84, v_1 = -1.2, v_2 = 18,\ v_3 = 12,\ v_4 = 17.4,$ and $\varphi = 0.0666667$. Other parameters are set as follows: $C = 20,\ g_{\mathrm{L,p}} = 0.06,\ g_{\mathrm{L},a} = 8, g_{\mathrm{Ca}} = 16,\ g_K = 32, g_{\mathrm{axial}} = 20,$ and $g_{\mathrm{gap}} = 3$.
